# Convergent approach to persistent atrial fibrillation ablation: long-term single-centre safety and efficacy

**DOI:** 10.3389/fcvm.2023.1336801

**Published:** 2024-02-08

**Authors:** Alexander Carpenter, Laura M. K. Pannell, Syed I. A. Rizvi, Kirsty Maciver, Cha Rajakaruna, Franco Ciulli, Edward R. Duncan, Glyn Thomas, Palash Barman, Richard Bond, Ashley M. Nisbet

**Affiliations:** ^1^ Bristol Heart Institute, Bristol, United Kingdom; ^2^ Departments of Physiology, Pharmacology, and Neuroscience, University of Bristol, Bristol, United Kingdom

**Keywords:** hybrid, convergent, atrial fibrillation, ablation, AF

## Abstract

**Background:**

Efforts to maintain sinus rhythm in patients with persistent atrial fibrillation (PsAF) remain challenging, with suboptimal long-term outcomes.

**Methods:**

All patients undergoing convergent PsAF ablation at our centre were retrospectively analysed. The Atricure Epi-Sense® system was used to perform surgical radiofrequency ablation of the LA posterior wall followed by endocardial ablation.

**Results:**

A total of 24 patients underwent convergent PsAF ablation, and 21 (84%) of them were male with a median age of 63. Twelve (50%) patients were obese. In total, 71% of patients had a severely dilated left atrium, and the majority (63%) had preserved left ventricular function. All were longstanding persistent. Eighteen (75%) patients had an AF duration of >2 years. There were no endocardial procedure complications. At 36 months, all patients were alive with no new stroke/transient ischaemic attack (TIA). Freedom from documented AF at 3, 6, 12, 18, 24, and 36 months was 83%, 78%, 74%, 74%, 74%, and 61%, respectively. There were no major surgical complications, with five minor complications recorded comprising minor wound infection, pericarditic pain, and hernia.

**Conclusions:**

Our data suggest that convergent AF ablation is effective with excellent immediate and long-term safety outcomes in a real-world cohort of patients with a significant duration of AF and evidence of established atrial remodelling. Convergent AF ablation appears to offer a safe and effective option for those who are unlikely to benefit from existing therapeutic strategies for maintaining sinus rhythm, and further evaluation of this exciting technique is warranted. Our cohort is unique within the published literature both in terms of length of follow-up and very low rate of adverse events.

## What’s new?

• Successful maintenance of sinus rhythm in patients with longstanding persistent atrial fibrillation (AF) remains a significant challenge, with endocardial catheter ablation often suboptimal.
• Convergent AF ablation combines surgical epicardial ablation of the left atrial posterior wall followed by endocardial pulmonary vein isolation.
• Our single-centre real-world data show favourable rates of maintenance of sinus rhythm at 36 months, which is a longer follow-up period when compared with existing studies.
• Importantly, we describe excellent safety outcomes without major complications or mortality in our cohort.
• Convergent AF ablation could be a safe and effective option for those likely to have limited success with conventional endocardial ablation strategies.

## Background

Catheter ablation (CA) of atrial fibrillation (AF) has been demonstrated to improve quality of life and symptoms in those with symptomatic AF (1–5). However, ablation of persistent AF (PsAF, defined as AF of continuous duration greater than 7 days) fares significantly worse in terms of medium- and long-term maintenance of sinus rhythm (6–8), likely related to progressive adverse remodelling of the atrial substrate (9).

Convergent (also known as “hybrid”) AF ablation involves a combination of surgical epicardial and endocardial catheter ablation to deliver durable pulmonary vein isolation (PVI). A minimally invasive thoracoscopic approach allows epicardial delivery of transmural left atrial (LA) posterior wall ablation. Subsequent endocardial studies are then used to confirm posterior wall isolation and further radiofrequency endocardial lesions delivered to complete PVI. Though relatively new with few published studies, it seems to offer significant promise in treating PsAF (10–13), although a significant increase in periprocedural complications is reported compared with endocardial CA alone (10, 12–14).

We undertook a single-centre retrospective observational study to investigate the safety, efficacy, and associated patient experience of the convergent ablation procedure to treat PsAF.

## Methods

The patients included in the study included all those undergoing the procedure from its inception locally and for a period of 18 months at our institution, a tertiary referral centre with electrophysiology and cardiac surgical expertise. Demographic, clinical, echocardiographic, and procedural data were collected. All patients undergoing convergent ablation over the time period were included. Left atrial (LA) and left ventricular (LV) echocardiographic data were recorded. LA dilatation was quantified by volume as non-dilated (≤ 58 ml), mildly dilated (59–68 ml), moderately dilated (69–78 ml), or severely dilated (≥ 79 ml). LV systolic function was assessed by Simpson's biplane ejection fraction (EF) using transthoracic echocardiography within 1 year preceding ablation.

Safety outcomes including perioperative complications, death, and stroke/transient ischaemic attack (TIA), as well as efficacy outcomes including freedom from documented or symptomatic AF, were collected at 3, 6, 12, 18, 24, and 36 months.

Obesity was defined as a body mass index (BMI) of ≥ 30. A positive smoking history was recorded as ≥20 smoking pack-years. The symptom burden was quantified using the AF symptom score established by the European Heart Rhythm Association (EHRA).

### Surgical epicardial ablation

Surgical ablation was performed under general anaesthesia and by one of two designated experienced cardiac surgeons with training in the procedure. Abdominal incisions and insufflation allowed access to the pericardium via the central tendon of the diaphragm. A pericardial window was created to allow access via a port to the posterior pericardium, and a 5 mm endoscope was introduced to visualise the posterior wall of the LA from left to right pulmonary veins. The Epi-Sense® Coagulation (Atricure® Inc, USA) ablation device was used to deliver lesions to the posterior left atrial wall under direct endoscopic vision. The transmurality of lesions was judged using preset energy delivery and impedance drop targets (> 10% from baseline). The sensing port of the Epi-Sense® device was used to confirm local electrical isolation and informed procedure completion. Continuous pericardial irrigation and oesophageal temperature monitoring were undertaken throughout lesion delivery. All patients received direct-current cardioversion at the end of the procedure.

### Endocardial catheter ablation

Catheter ablation was undertaken by experienced operators via femoral venous access, 6–12 weeks following surgical ablation. Either local or general anaesthesia was used according to individual patient factors and patient preference. Anticoagulation was uninterrupted. Trans-septal puncture was fluoroscopically guided, using trans-oesophageal (TOE) guidance where necessary. Electro-anatomical mapping of the LA was generated using the CARTO® software package (Biosense Webster, USA). Ablation consisted of PVI via a wide area circumferential ablation (WACA) strategy to isolate the pulmonary veins. Where required, further ablation including roof and/or floor lines, and more extensive ablation (complex fractionated atrial electrograms, mitral/tricuspid isthmus or coronary sinus ablation) was undertaken to achieve PV isolation. Where posterior LA wall isolation was incomplete as demonstrated by voltage mapping, these gaps were addressed with additional ablation and posterior wall isolation confirmed. PV isolation was confirmed by pacing to confirm the entrance and exit block. SmartTouch® (Biosense Webster, USA) contact-force sensing ablation catheters were used, and the force–time integral guided the lesion delivery.

### Follow-up

Patients were seen in an outpatient setting at 3, 6, and 12 months following their final endocardial ablation procedure, during which an office electrocardiogram (ECG) was performed. Further follow-up at 18, 24, and 36 months occurred either locally, in primary care, or at the referring local hospital. Further AF monitoring comprised symptom-led Holter recording at any point during follow-up. A team of specialised arrhythmia nurses facilitated enhanced patient education and provided a responsive and accessible point of contact for patients throughout the process.

Longstanding PsAF was defined as those suffering with AF episodes lasting for >1 year. AF freedom was defined as the absence of documented AF by office 12-lead ECG testing at follow-up. ECG AF documentation in primary care records or direct patient communication was included.

### Statistical analysis

Statistical analysis was performed using GraphPad PRISM (GraphPad Software, USA). Data are presented as median ± interquartile range.

The study was approved by the institutional clinical audit and research department and received NHS Health Research Authority (HRA) approval.

## Results

### Baseline characteristics

Over an 18-month period, 24 patients underwent the convergent ablation procedure for longstanding PsAF. Patient baseline characteristics and arrhythmia data are displayed in Table 1. In total, 84% (*n* = 21) of patients were male, with a median age of 63 and median BMI of 30. A total of 36% (*n* = 11) of patients suffered from hypertension, 17% (*n* = 4) patients suffered from obstructive sleep apnoea (OSA), and 8% (*n* = 2) of patients suffered from diabetes. A total of 38% (*n* = 9) of patients exhibited a significant smoking history, and the median alcohol units consumed per week was 14. The median CHA_2_DS_2_VASc score was 1, with 71% (*n* = 17) of patients falling in the 0–1 bracket.

**Table 1:** Baseline characteristics and arrhythmia data of patients with atrial fibrillation undergoing convergent ablation procedure.

Baseline characteristics	All patients, [*N* (%)] *n* = 24
Age	
Median (IQR)	63 (10) Range 42–71
<65	16 (67%)
65–74	8 (33%)
≥75	0
Male gender	21 (84%)
BMI	
Median (IQR)	30 (9) Range 26–41
≥30	12 (50%)
Hypertension	11 (46%)
Obstructive sleep apnoea	4 (17%)
Diabetes	2 (8%)
Smoking history (≥20 pack-years)	9 (38%)
Alcohol units per week [median (IQR)]	14 (16)
CHA_2_DS_2_VASc	
Median (IQR)	1 (2)
0–1	17 (71%)
2	5 (21%)
3	2 (8%)
4	0
≥5	0
Left atrial volume, ml [median (IQR)]	
Median (IQR)	99 (34)
Non-dilated, ≤58 ml	3 (13%)
Mildly dilated, 59–68 ml	2 (8%)
Moderately dilated, 69–78 ml	2 (8%)
Severely dilated, ≥79 ml	17 (71%)
Left ventricular ejection fraction (%)	
Mean (SD)	52 (6)
≥50%	15 (63%)
40%–49%	6 (25%)
<40%	3 (13%)
Arrhythmia history	
Duration of AF	
<1 year	0
1–2 years	6 (25%)
>2 years	18 (75%)
EHRA symptom score	
1 (none)	0
2a (mild)	0
2b (moderate)	20 (83%)
3 (severe)	4 (17%)
4 (disabling)	0
Symptom reported	
Breathlessness	18 (75%)
Fatigue/lethargy	16 (67%)
Reduced exercise tolerance	3 (13%)
Prior AF catheter ablation	
1 prior ablation	1 (4%)
≥2 prior ablations	1 (4%)
Rhythm-control medication	2 (8%)
Anticoagulation	
DOAC	20 (83%)
Warfarin	4 (17%)

IQR, interquartile range; BMI, body mass index; SD, standard deviation; AF, atrial fibrillation; EHRA, European Heart Rhythm Association; DOAC, direct oral anticoagulant.

The majority of patients (71%; *n* = 17) had a severely dilated LA. The majority (63%; *n* = 15) of patients had preserved LV function (≥ 50%), 25% (*n* = 6) had mild impaired function (LVEF 40%–49%), and 13% (*n* = 3) suffering from significantly impaired function (LVEF < 40%), consistent with the 2016 ESC heart failure classification (15).

### Arrhythmia data

All patients had longstanding PsAF, with AF duration exceeding 1 year, with 75% (*n* = 18) greater than 2 years, and 25% between 1 and 2 years. A total of 83% (*n* = 20) of patients had a “moderately disabling” EHRA symptom score, with 17% (*n* = 4) of patients having a “severely disabling” score. A total of 75% (*n* = 18) of patients reported breathlessness as a symptom, 67% (*n* = 16) reported fatigue or lethargy, and 13% (*n* = 3) reported reduced exercise tolerance. The observed overlap reflects the frequent multiple symptom burden in patients. Two patients had previously undergone AF catheter ablation, one of whom had undergone a single ablation and the other patient underwent three previous ablations. In total, 8% (*n* = 2) of patients were on rhythm-controlling medications at the time of work-up (flecainide, amiodarone). All patients were anticoagulated, with 83% (*n* = 20) of them on a direct oral anticoagulant (DOAC) and the remaining patients taking warfarin.

### Procedural and outcome data

Full data are shown in Table 2. The median number of surgical lesions delivered was 25 (interquartile range of 12) with a minimum number of 13 lesions and a maximum of 41 lesions. The average number of lesions delivered seemed to increase over the study period: the first 12 patients had a mean of 21.6 lesions while the latter 12 patients had a mean of 26 lesions. However, this did not seem to correlate with any clear change in efficacy. The patients stayed in the hospital for a median of 2 days.

**Table 2:** Procedural and outcome data of patients with atrial fibrillation undergoing convergent ablation procedure.

Procedural data	All patients, [*N* (%)] *n* = 24
No. surgical lesions delivered [median (IQR)]	25 (12) Range 13–41
Surgical inpatient days [median (IQR)]	2 (0.25)
Endocardial ablation strategy	
PVI only	5 (21%)
PVI + roof line	5 (21%)
PVI, roof line and floor	10 (42%)
PVI, roof, floor plus additional (CFAEs, mitral or cavo-tricuspid isthmus and/or CS)	4 (17%)
Further re-do endocardial procedures undertaken	
1 within 12 months for AF	4 (17%)
≥2 within 12 months for AF	0
Within 12 months for atrial tachycardia	1 (4%)
Outcome data	
Freedom from documented AF	
At 3 months	20/24 (83%)
At 6 months	18/23 (78%)
At 12 months	17/23 (74%)
At 18 months	17/23 (74%)
At 24 months	17/23 (74%)
At 36 months	14/23 (61%)
Safety data	
Mortality	
At 3 months	0
At 6 months	0
At 12 months	0
At 18 months	0
At 24 months	0
At 36 months	0
New diagnosis stroke/TIA	
At 3 months	0
At 6 months	0
At 12 months	0
At 18 months	0 (of 23 under follow-up)
At 24 months	0 (of 23 under follow-up)
At 36 months	0 (of 23 under follow-up)
Periprocedural complications	
Endocardial procedure complications	0
Post-surgical minor wound infection	3 (13%)
Admission for post-surgical pericarditic pain	1 (4%)
Post-surgical incisional hernia	1 (4%)
Significant bleeding	0
Pericardial effusion	0
Infective endocarditis	0
Other	0

Data are shown for procedural outcomes. IQR, interquartile range; PVI, pulmonary vein isolation; CFAE, complex fractionated atrial electrogram; CS, coronary sinus; AF, atrial fibrillation; TIA, transient ischaemic attack.

**Figure 1:**
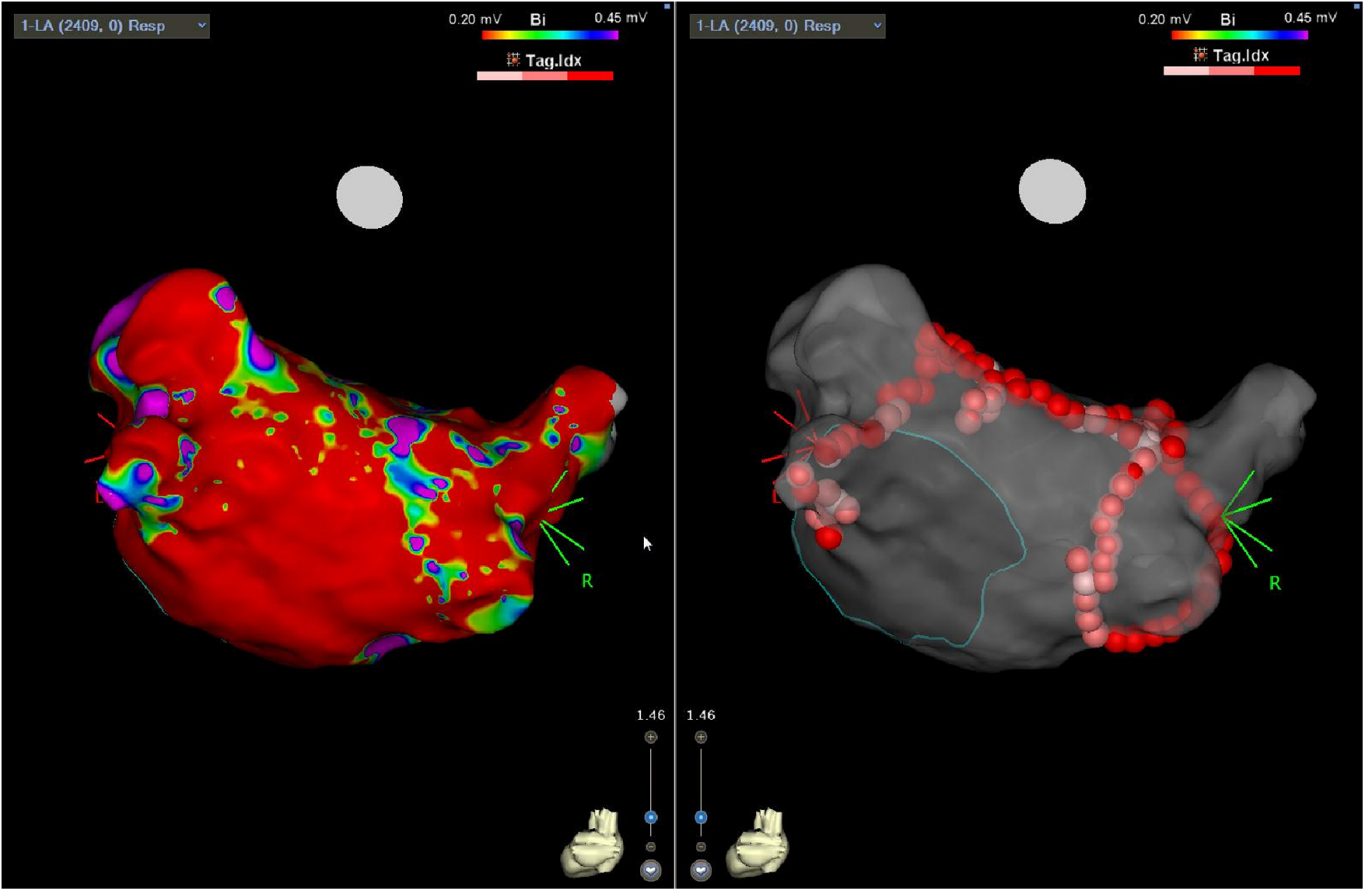
Three-dimensional electro-anatomical map showing posterior LA with diffuse regions of low voltage (left panel) following surgical epicardial ablation and subsequent endocardial PVI (right panel).

The endocardial ablation strategies used included PVI only (21%; *n* = 5), PVI plus roof line (21%, *n* = 5), PVI, roof line, and floor (42%, *n* = 10), and more extensive ablation (complex fractionated atrial electrograms, mitral/tricuspid isthmus or coronary sinus ablation) in 17% (*n* = 4) of patients. Figure 1 demonstrates a representative 3D anatomical left atrial voltage map showing the extensive scar on the posterior wall, as well as the subsequent endocardial ablation undertaken to complete the PVI.

Following the surgical ablation procedure, there were three (13%) minor wound infections, all treated successfully with oral antibiotics in the outpatient setting. There was a single occurrence of re-admission for postoperative pericarditic pain, and a single occurrence of a post-surgical incisional hernia. There were no major complications, significant pericardial effusion, bleeding, or infective endocarditis. There were no immediate endocardial catheter ablation complications. There was no post-procedural mortality.

There was no mortality recorded within the study population at 3, 6, 12, 18, 24, or 36-month follow-up. Similarly, there were no new diagnoses of stroke/TIA at 3, 6, 12, 18, 24, or 36-month follow-up.

Further re-do catheter ablation for AF was undertaken in four patients (17%), all undergoing a single further procedure within 12 months. Separately, one patient (4%) underwent a further catheter ablation procedure within 12 months for symptomatic atrial tachycardia.

**Figure 2:**
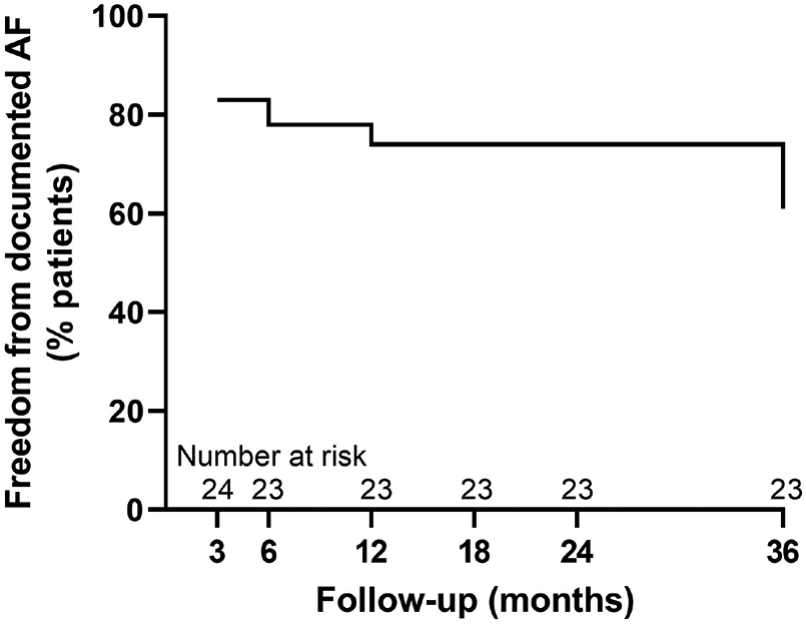
Survival plot showing single-procedure freedom from documented AF at 3-, 6-, 12-, 18-, 24- and 36-month follow-up.

The freedom from documented AF was 83% (*n* = 20/24) at 3 months, 78% (*n* = 18/23) at 6 months, 74% (*n* = 17/23) at 12 months, 74% (*n* = 17/23) at 18 months, 74% (*n* = 17/23) at 24 months, and 61% (*n* = 14/23) at 36 months. The survival plot is illustrated in Figure 2.

One patient was lost to follow-up before 18 months due to disruption related to the COVID-19 pandemic, although the mortality data were confirmed for all 24 patients.

## Discussion

### Procedural outcomes and safety

A convergent approach to AF ablation has been described in several small studies (11, 13, 14, 16–19) as well as a recent meta-analysis (10) and randomised controlled trial (RCT) (12). They report very favourable rates of maintenance of sinus rhythm in the medium and long term. However, they also report a significant burden of periprocedural complications with associated morbidity and mortality. Our small study shows that the convergent ablation approach can be undertaken safely. Our cohort found a low number of minor procedural complications, which consisted of wound infections, pericarditic pain, and a postoperative incisional hernia. All wound infections were successfully treated with oral antibiotics in the outpatient setting. The number of surgical lesions delivered seemed to increase throughout the study period, although without observable effect on efficacy.

Importantly, and in contrast to other reported studies, there were no major periprocedural complications and no stroke, TIA, or mortality observed periprocedurally or during follow-up. Thus, despite the findings of the recent RCT (12) and other published reports with larger patient cohorts, our data are novel both in terms of the very low rate of serious periprocedural complications and the follow-up data—longer than any other published cohort, at 3 years.

### Arrhythmia-free survival and quality of life

The rates of maintenance of sinus rhythm are favourable and in keeping with other published studies (1, 20) with 78% of patients maintaining sinus rhythm at 6 months, 74% at 12, 18, and 24 months, and 61% at 3 years, in a cohort of patients with longstanding PsAF and evidence of established remodelling.

There were a small number of re-do endocardial procedures within the study period (four re-do procedures for AF, one for focal atrial tachycardia). The use of repeat ablation procedures is common in real-world practise to promote sinus rhythm (21), allowing assessment of the PVI and further ablation to be delivered if required to address gaps, promoting durable block.

Our 36-month duration of long-term follow-up is unique within the literature and provides a useful insight into the long-term disease course of these patients.

### Convergent AF ablation and arrhythmia mechanisms

Our study captures a cohort of patients undergoing ablation for longstanding PsAF which reflects real-life practise, with a high burden of reported symptoms and a significant duration of AF. Our cohort also reflects a group of patients with typical upstream AF disease drivers with significant rates of obesity, hypertension, and diabetes. These are individuals who are likely to experience a poor long-term outcome from conventional endocardial PsAF catheter ablation (6–8), and in whom satisfactory rhythm control for AF remains an ongoing unmet need. Indeed, 75% of patients in our cohort had an AF duration of greater than 2 years, and 71% of patients had a severely dilated LA. These individuals are likely to demonstrate established atrial remodelling and fibrosis. The mechanisms of failure of AF ablation in these types of individuals remain unclear, with increased difficulty in obtaining durable isolation of both PV (22, 23) and non-PV AF triggers (3, 24) likely playing a role. Indeed, repeated endocardial ablation procedures may indeed generate new non-PV triggers (23, 25), thus exacerbating the situation. However, extensive ablation in pursuit of such foci has failed to show any compelling benefit beyond PVI alone (7).

Convergent ablation offers the potential for durable posterior LA wall ablation. This is then confirmed at the time of endocardial ablation, and completed if required. PVI is then undertaken, at which point further ablation is performed if felt beneficial. This more extensive ablation lesion set may potentially recruit more PV as well as non-PV triggers, as there has been a suggestion that the posterior LA wall, and in particular localised atrial fat, may play an important role in persistent AF (26, 27). In addition, there may be some endocardial–epicardial dissociation contributing to arrhythmogenesis in those with persistent AF (28, 29), which is more effectively targeted by a convergent approach. The advent of novel ablation technologies such as pulsed field ablation (PFA) presents further opportunities for convergent AF ablation and merits further study.

### Limitations

Our data are primarily limited by a lack of quantitative rhythm monitoring. The use of continuous rhythm monitoring such as with an implantable loop recorder (ILR) would have enhanced two aspects of the study: first, it would have enabled an accurate assessment of AF recurrence. Second, it would have allowed a quantitative assessment of AF burden before and after ablation. Indeed, the assessment of arrhythmia (as well as symptom) burden may be more important than binary absolute recurrence. A further limitation of the data was lack of consistently available procedural data as well as LA dwell time.

The low number of patients taking rhythm-control anti-arrhythmic medication may not be representative of the universal experience of patients with longstanding PsAF. However, it may be that medication intolerance or lack of efficacy was an influencing factor in the decision to pursue convergent ablation.

## Conclusions

Convergent ablation offers favourable short- and long-term efficacy. Our study is the first to show that it can be undertaken safely without significant complications or mortality and with durable efficacy as far out as 36 months. As the technique continues to develop and experience with the procedure grows, we may see continued improvements in efficacy and safety.

Convergent ablation for persistent AF offers an attractive option for a patient group suffering with an atrial substate that has already undergone advanced remodelling. They are not well served by existing conventional endocardial ablation and this continues to represent an unmet need in a cohort with a high symptom burden. This new technique offers an exciting new approach to improve quality of life and would be well served by further comprehensive evaluation.

## Data Availability

The raw data supporting the conclusions of this article will be made available by the authors, where appropriate without undue reservation.
